# Endolysosomal Cation Channels and Cancer—A Link with Great Potential

**DOI:** 10.3390/ph11010004

**Published:** 2018-01-05

**Authors:** Christian Grimm, Karin Bartel, Angelika M. Vollmar, Martin Biel

**Affiliations:** 1 Munich Center for Integrated Protein Science CIPSM, 81377 München, Germany; 2 Department of Pharmacy, Center for Drug Research, Ludwig-Maximilians-Universität München, 81377 München, Germany; karin.bartel@cup.uni-muenchen.de

**Keywords:** calcium, TPC, two-pore, lysosome, TPC1, TPC2, TRPML, mucolipin, MCOLN, mTOR, TRPML1, TFEB

## Abstract

The endolysosomal system (ES) consists of lysosomes; early, late, and recycling endosomes; and autophagosomes. It is a key regulator not only of macromolecule degradation and recycling, plasma membrane repair, homeostasis, and lipid storage, but also of antigen presentation, immune defense, cell motility, cell death signaling, tumor growth, and cancer progression. In addition, it plays a critical role in autophagy, and the autophagy-lysosome pathway is intimately associated with the hallmarks of cancer, such as escaping cell death pathways, evading immune surveillance, and deregulating metabolism. The function of endolysosomes is critically dependent on both soluble and endolysosomal membrane proteins such as ion channels and transporters. Cation channels found in the ES include members of the TRP (transient receptor potential) channel superfamily, namely TRPML channels (mucolipins) as well as two-pore channels (TPCs). In recent studies, these channels have been found to play crucial roles in endolysosomal trafficking, lysosomal exocytosis, and autophagy. Mutation or loss of these channel proteins can impact multiple endolysosomal trafficking pathways. A role for TPCs in cancer cell migration and metastasis, linked to distinct defects in endolysosomal trafficking such as integrin trafficking, has been recently established. In this review, we give an overview on the function of lysosomes in cancer with a particular focus on the roles which TPCs and TRPML channels play in the ES and how this can affect cancer cells.

## 1. Introduction

In Europe and the USA, cancer and cancer-related diseases account for about 25–30% of deaths. Curative therapies are still the exception. Hence, there is an urgent need to find novel, innovative targets and therapies and to develop new treatment strategies.

Targeting the lysosome has emerged as an increasingly attractive strategy in cancer therapy in recent years [1,2,3]. That links exist between cancer and lysosomal function is not new. It has already been postulated decades ago that an increased liberation of lysosomal hydrolases in tumors could contribute to inflammatory and toxic effects and could promote the detachment of cells from tumor masses and thus facilitate metastatic spread. In addition, several studies at the time had revealed higher activities of different lysosomal enzymes in solid tumors than in their tissues of origin [4,5,6]. Today, critical functions of the lysosome in cancer cell biology as well as differences in cancer cell lysosomes versus normal lysosomes are widely recognized. How exactly lysosomes contribute to tumorigenesis and cancer progression is, however, still being uncovered [1,7].

Besides the lysosome-cancer link, there are also many established connections between cancer and ion channels, both ion channels on the plasma membrane and on intracellular membranes. The oncogenic intracellular ion channels comprise mitochondrial channels such as Kv1.3 (KCNA3), IK_Ca_ (KCNN4), and TASK-3, among others, in addition to the intracellular chloride channel CLIC-4 or different TRP (transient receptor potential) channels, e.g., TRPM8 and TRPC1 (for excellent recent reviews see e.g., [8,9]; for other TRP channels in relation to cancer see e.g., [10,11]). Very recently, the calcium permeable, non-selective endolysosomal two-pore cation channels (TPCs) were found to play a role in cancer cell migration and β1-integrin trafficking and recycling [12,13]. In the following, we give an overview on the roles of lysosomes in cancer and the involvement of endolysosomal TRPML channels and TPCs.

## 2. Cancer Cell Lysosomes

Lysosomes in cancer cells are different from normal lysosomes in several aspects. For example, enhanced protease activity and release from cancer cell lysosomes into the extracellular space is often observed and found to promote tumor progression [3,14,15,16]. Secretion of cathepsins into the extracellular space seems to be facilitated by altered trafficking of lysosomes in cancer cells, which results in a shift of localization from perinuclear to peripheral. By contrast, cytosolic release of lysosomal enzymes has been demonstrated to trigger apoptosis and cell death, providing a rational for cancer therapies aiming at the destabilization of the lysosomal membranes [17,18]. Cancer cells need plenty of nutrients to grow. This leads to lysosomal alterations in cancer cells such as increased expression and altered trafficking of lysosomal enzymes, as mentioned above. Similarly, alterations in the autophagic compartment are linked to carcinogenesis as well as resistance to chemotherapy. For example, the signaling pathways that activate mammalian target of rapamycin (mTOR) are altered in many human cancers [19]. mTOR is a key signaling regulator of autophagy, and the inhibition of mTOR by e.g., rapamycin or nutrient deprivation, induces the activation of autophagy [20,21]. mTOR is also a downstream effector of the PI3K-AKT pathway. PI3K (phosphatidylinositol-4,5-bisphosphate-3-kinase), which regulates the maturation, size, and content of the lysosomal compartment, shows increased activity in many cancers [22,23]. In particular, the PI3K-AKT-mTOR cascade is frequently hyperactivated in cancer, and plays an integral role in tumor growth and survival [24]. Furthermore, the phosphatase that negatively regulates PI3K, the tumor suppressor PTEN (phosphatase and tensin homolog) is mutated, silenced, or deleted in a number of tumor types including glioblastoma, lung carcinoma, melanoma, hepatocellular carcinoma, and prostate cancer [19].

The activation of PI3K can occur through tyrosine kinase growth factor receptors such as epidermal growth factor receptor (EGFR) and insulin-like growth factor-1 receptor (IGF-1R), cell adhesion molecules such as integrins, G-protein-coupled receptors (GPCRs), and oncogenes such as Ras [25]. The receptors that function upstream of PI3K are often mutationally activated or overexpressed in cancer. Interfering with lysosomal function by the inhibition of the vacuolar H^+^-ATPase, which is essential for lysosomal acidification, was recently demonstrated to abrogate excessive EGFR and Ras signaling in cancer cells, leading to reduced migration and proliferation [26,27]. These findings underline the potential of cancer cell lysosomes as drug targets. Finally, the subcellular positioning (e.g., peripheral versus perinuclear) and distribution of lysosomes seems to contribute to various pathologies including cancer progression [28,29].

## 3. Cancer and Inflammation

Inflammation is considered to be a generic mechanism of innate immunity as compared to adaptive immunity, which is specific for each pathogen. The process of acute inflammation is initiated by resident immune cells already present in the involved tissue, mainly resident macrophages, dendritic cells, Kupffer cells, and mast cells. Chronic inflammation is caused by a variety of factors, including bacterial, viral, and parasitic infections, chemical irritants, and non-digestible particles. That chronic inflammation can increase the risk of cancer and other diseases is now widely accepted. Several pathologies illustrate this link, such as endometriosis, chronic prostatitis, chronic gastritis, or inflammatory bowel disease (IBD) [30]. In IBD, especially Crohn’s disease patients, increased numbers of cells positive for interferon-γ (IFN-γ), which is a proinflammatory cytokine with multiple functions, have been observed, possibly contributing to a chronic inflammatory setting [30,31]. If inflammation persists, the risk for associated carcinogenesis is increased [32]. Cancer cells also use proinflammatory chemokines and their receptors for invasion, migration, and metastasis [33]. On the other hand, many cells of the immune system also contribute to immune surveillance of cancer and cancer suppression [33]. How this critical balance between the positive and negative effects of inflammation and immune response is maintained and how a negative shift in this balance is promoted in cancer progression needs to be further elucidated.

In recent years more and more evidence has accumulated demonstrating that TRPML channels and TPCs are expressed in cells of the immune system, in particular macrophages, mast cells, and dendritic cells, and that they may regulate the production and secretion of inflammatory mediators but also immune cell migration [34,35,36]. In addition, TRPMLs and TPCs have been found to be involved in mTOR signaling, autophagy, and EGF/EGFR as well as integrin trafficking.

## 4. TPCs and Cancer

TPCs are found in early endosomes as well as late endosomes/lysosomes and are postulated to participate in the regulation of intracellular trafficking and fusion processes.

Links between TPCs and cancer have been summarized in a recent review by Parrington et al. (2015) [37]. In addition, Nguyen et al. [13] have recently shown that TPCs play a crucial role in cancer cell migration and tumor cell dissemination, as silencing TPC1 and TPC2 with siRNA or pharmacological inhibition reduces the adhesion and migration of invasive tumor cells and the formation of lung metastases in an in vivo mouse model. Endosomes and lysosomes control integrin trafficking and recycling which is required for (cancer) cell migration [28,38,39]. The inhibition of TPCs leads to an accumulation of β1-integrin in endocytic vesicles and to an impaired lamellipodia formation, indicating that TPCs are significantly involved in integrin recycling and directed migratory processes.

Knockout mice lacking TPC2 also show an increased intracellular accumulation of EGF/EGFR, potentially resulting from a defect in EGF/EGFR trafficking to lysosomes and subsequent degradation [40]. This disrupted degradation of EGF/EGFR could lead to prolonged EGFR signaling in endosomes, which may impact cancer cell proliferation. In addition, EGFR recycling may be affected or changes in EGFR signaling may impact the PI3K-AKT-mTOR cascade and thus tumor growth and survival, as discussed above.

It was also shown recently that the blockade of TPCs inhibits VEGF-induced neoangiogenesis (formation of new blood vessels) [41] and that VEGF-induced neoangiogenesis is mediated by NAADP and TPC2-dependent calcium signaling [42]. Neoangiogenesis accompanies tissue regeneration and healing, but is also crucial for tumor growth [42]. As Favia et al. [42] pointed out, the formation of new vascular capillaries, e.g., in inflammatory or cancer processes, proceeds through a defined sequence of steps as diverse as cell proliferation, migration, differentiation, and morphogenesis, all of which involve control by VEGF-linked signaling cascades. Favia et al. [42] further showed that the inhibition of signaling pathways involving VEGFR2, NAADP, TPC2, and Ca^2+^ release from acidic stores significantly decreases the activation of the known VEGFR2 downstream targets ERK1/2 MAPK, JNK, Akt, and eNOS, and blocks angiogenesis in both in vitro and in vivo models. Whether this is a major mechanism by which the blockade of TPCs results in anticancer effects awaits further exploration.

**Figure 1:**
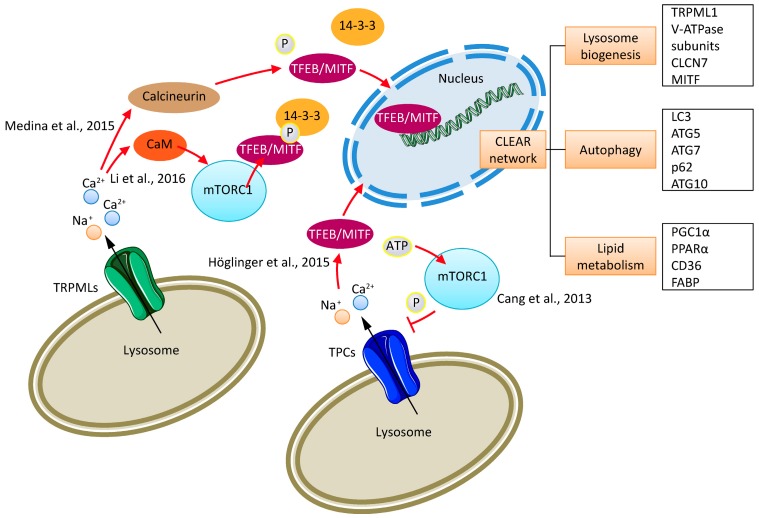
Endolysosomal cation channels and mTORC1. Shown is an illustration of the mechanisms underlying TFEB and mTORC1 modulation by lysosomal calcium released through TRPML1 and the physical association and functional regulation (inhibition) of TPC activity by mTORC1. Li et al. [45] reported the TRPML1-dependent activation of CaM, which associates with mTOR and activates mTORC1. mTORC1 phosphorylates TFEB. This is required for its interaction with 14-3-3 and the prevention of TFEB nuclear translocation. Medina et al. [46] described a TRPML1-dependent activation of calcineurin, which leads to the dephosphorylation of TFEB. TFEB then translocates to the nucleus to start the transcription of CLEAR network genes [47,48]. Furthermore, mTORC1 activation can block TPC activity and thus calcium/sodium flux from lysosomes (Cang et al. [43]). Sphingosine-induced activation of TPC1 leads to the translocation of TFEB to the nucleus after lysosomal calcium release (Höglinger et al. [49]).

Importantly, it was also found that TPCs directly interact with mTOR [43]. High ATP levels, such as those found in nutrient-replete cells, are postulated [43] to enable mTOR to phosphorylate TPCs and to thus maintain the channel in the closed state (Figure 1). The channel complex thus detects nutrient status and becomes constitutively open upon nutrient removal and mTOR translocation off the lysosomal membrane [43]. mTOR is an atypical serine/threonine kinase that is present in two distinct complexes. mTOR complex 1 (mTORC1) has five components: mTOR, which is the catalytic subunit of the complex; regulatory-associated protein of mTOR (Raptor); mammalian lethal with Sec13 protein 8 (mLST8, also known as GbL); proline rich AKT substrate 40 kDa (PRAS40); and DEP-domain-containing mTOR-interacting protein (Deptor) [44]. The second complex, mTOR complex 2 (mTORC2) comprises six different proteins, several of which are common to mTORC1 and mTORC2: mTOR; rapamycin-insensitive companion of mTOR (Rictor); mammalian stress-activated protein kinase interacting protein (mSIN1); protein observed with Rictor-1 (Protor-1); mLST8; and Deptor [44]. mTORC2 promotes cellular survival by activating AKT, regulates cytoskeletal dynamics by activating PKCα, and controls ion transport and growth via SGK1 phosphorylation.

Knockdown of Raptor, but not Rictor, reduced the ATP inhibition of TPCs, suggesting that the ATP sensitivity of TPCs is conferred by the mTORC1 complex [43].

It is well established that aberrant mTOR signaling is involved in many disease states including cancer, cardiovascular disease, and diabetes. How the interaction with and the inhibition by mTOR might affect possible roles of TPCs in cancer development and progression remains to be elucidated.

## 5. TRPMLs and Cancer

In contrast to TPCs, the ATP-insensitive endolysosomal TRPML channels were found to have little or no detectable association with mTOR [43]. Nevertheless, TRPML1 has recently been shown to be involved in mTOR signaling [45,46,50]. The blockade of lysosomal calcium release due to lysosomal lipid accumulation is known to inhibit mTOR signaling. The mechanism by which lysosomal calcium regulates mTOR remained undefined, until Li et al. [45] reported that proper lysosomal calcium release through TRPML1 is required for mTORC1 activation. Consequently, TRPML1 depletion inhibits mTORC1 activity, while the overexpression or pharmacologic activation of TRPML1 has the opposite effect. Lysosomal calcium activates mTORC1 by inducing the association of calmodulin (CaM) with mTOR (Figure 1). Blocking the interaction between mTOR and CaM by antagonists of CaM significantly inhibits mTORC1 activity. Moreover, CaM is capable of stimulating the kinase activity of mTORC1 in a calcium-dependent manner in vitro. The results provided by Li et al. [45] revealed that mTOR is a new type of CaM-dependent kinase, and TRPML1, lysosomal calcium, and CaM play essential regulatory roles in the mTORC1 signaling pathway [45].

Medina et al. [46] found that lysosomal calcium release through TRPML1 activates calcineurin, which binds and dephosphorylates transcription factor EB (TFEB), thus promoting its nuclear translocation. TFEB is a master regulator of lysosomal and autophagic function and, intriguingly, the induction of autophagy and lysosomal biogenesis through TFEB requires TRPML1-mediated calcineurin activation (Figure 1). Altered TFEB expression and/or activity has recently been associated with pancreatic cancer cell proliferation [51] and non-small cell lung cancer motility [52], and Calcagnì et al. recently showed that kidney-specific TFEB overexpression in transgenic mice resulted in renal clear cells, multi-layered basement membranes, severe cystic pathology, and ultimately papillary carcinomas with hepatic metastases [53].

As described by Roczniak-Ferguson et al. [54] TFEB is recruited to lysosomes via an interaction with mTORC1, and the mTORC1-dependent phosphorylation of TFEB is required for its interaction with 14-3-3 and the prevention of TFEB nuclear translocation. The exact mechanism(s) governing the TRPML1-mediated regulation of mTOR and TFEB signaling, as described by Li et al. [45] and Medina et al. [46], need further investigation. In this context, it is also of interest that TPC1 mediated endolysosomal calcium release by sphingosine (SPH), which has been postulated by Höglinger et al. [49] to be important for the translocation of TFEB to the nucleus [49] (Figure 1).

These findings illustrate that the lysosome is not only important for degradation and trafficking/recycling processes but is also discussed as a signaling hub affecting different transcription factors, in particular TFEB, with an important impact on metabolism and cancer [55,56].

Following the hypothesis that TRPMLs and TPCs play a role in mTOR signaling by affecting MITF/TFEB activity and thus the CLEAR gene network (coordinated lysosomal expression and regulation gene network; [47,48]), a more detailed analysis of TFEB activation (i.e., translocation into the nucleus) and the transcription of TFEB target genes in WT versus TRPML/TPC KD/KO cancer cells is warranted. Beyond that, other aspects of TRPML channel activation such as the impact of TRPML1 on lysosomal exocytosis [57,58,59] or lysosome motility [60] need to be studied in more detail in the context of cancer. Namely, the inhibition of lysosomal exocytosis has been recently demonstrated to reverse invasiveness and chemoresistance in aggressive sarcoma cells, revealing that lysosomal exocytosis plays a primary role in tumor progression and chemoresistance [61]. Furthermore, TRPML2 knockdown has recently been shown to inhibit the viability, to alter the cell cycle, to reduce the proliferation, and to induce apoptotic cell death in glioma cell lines. The high TRPML2 expression levels in glioma cells resulted in increased survival and proliferation signaling, suggesting a pro-tumorigenic role played by TRPML2 in glioma progression [62].

In summary, it is evident that TPCs and TRPMLs are functionally relevant at the key control points of intracellular trafficking and transport, affecting cell proliferation, autophagy, and survival. This is specifically highlighted by their interference with mTOR and TFEB function and signaling.
